# Telocytes transfer extracellular vesicles loaded with microRNAs to stem cells

**DOI:** 10.1111/jcmm.12529

**Published:** 2015-01-20

**Authors:** Valeriu B Cismasiu, Laurentiu M Popescu

**Affiliations:** a Laboratory of Cellular Medicine, “Victor Babeş” National Institute of PathologyBucharest, Romania; b Department of Cellular and Molecular Medicine, “Carol Davila” University of MedicineBucharest, Romania; c Division of Advanced Studies, National Institute of PathologyBucharest, Romania

**Keywords:** extracellular vesicles, cardiac stem cells, hematopoietic stem cells, telocytes, microRNAs, miR mimic

## Abstract

Telocytes (TCs) are cells ubiquitously distributed in the body and characterized by very long and thin prolongations named telopodes (Tps). Cardiac TCs are the best characterized TCs for the moment. Tps release extracellular vesicles (EVs) *in vivo* and *in vitro* suggesting that TCs regulate the activity of other cells by vesicular paracrine signals. TCs have been found within the stem cell niche of several organs. Electron microscopy or electron tomography has shown that Tps are located in close vicinity of stem cells (SC). Since stem cell regulation by niche components involves paracrine signalling, we have investigated if TCs could be part of this mechanism. Using fluorescent labelling of cells and EVs with calcein and Cy5-miR-21 oligos, we provide evidence that TCs can modulate SC through EVs loaded with microRNAs. TCs deliver microRNA to cardiac stem cells (CSCs), as well as to other types of SCs (*e.g*. hematopoietic SC) indicating that this mechanism is not restricted to cardiac tissue. We also found that CSCs deliver microRNA loaded EVs to TCs, suggesting that there is a continuous, post-transcriptional regulatory signal back and forth between TCs and SC. In conclusion, our data reveal the existence of a reciprocal (bidirectional) epigenetic signalling between TCs and SC.

## Introduction

Stem cell populations are located in ‘niches’ – specific anatomic sites that regulate how they participate in tissue generation, maintenance and repair 1. The niche includes all cellular and non-cellular components that interact to control the adult stem cell, and these interactions can often be broken down into one of two major mechanistic categories: physical contacts and diffusible factors 2. One component of paracrine signalling is represented by specialized subtypes of the extracellular vesicles (EVs), called exosomes and microvesicles 3,4. Accumulating evidence suggests that both types of EVs can shuttle genetic material (*i.e*. mRNAs and small, single strand RNAs called microRNAs or miRs) between cells over a long distance and it has been shown that the translocated miRs can modulate gene expression and the phenotype of the recipient cells 5–7. The mechanism may be important in cardiac regeneration and tissue remodelling 7,8. Thus, it has been shown that post-mytotic cardiomyocytes deliver miR-499 to the cardiac stem cells (CSCs) that in turn promote CSC differentiation 9.

Telocytes (TCs) are interstitial cells previously characterized by electron microscopy as cells with very long extensions (telopodes, Tps) 10–12. TCs have several morphological and phenotypical properties 13 as well as a specific gene expression 14–16 and proteomic profiles 17,18 that define TCs as a distinct type of interstitial cells. The most advanced morphological studies using focused ion beam scanning electron microscopy (FIB-SEM tomography) showed the 3D reconstruction of cardiac TCs 19. Given that TCs interact with identical cells through Tps (homojunctions) generating a network as well as with other type of cells (heterojunctions) including vascular and nervous cells it has been suggested that TCs have a role in the fine-tuning of regulatory signals delivered through vascular, nervous and endocrine systems 20. Thus, the TC network within the tissue stroma ensures that regulatory signals are distributed coherently within the tissue mass. While the biological function of TCs is not fully understood, TCs have been found in stem niche in the vicinity of cardiac stem and progenitor cells 12,20,21. Moreover, TCs were (always) found in stem cell niches described by EM and ET in lung 22, bone marrow 23, dermis 24, eye 25, meninges and choroid plexus 26. EVs were found in the proximity of TCs in cardiac tissue after experimental myocardial infarction 27. A recent review is available 28.

We have shown recently that TCs release three types o EVs in culture 29. Under the working hypothesis that TCs modulate stem cells by paracrine signals, we show here that TCs deliver microRNAs to CSCs through EVs. Reciprocally, CSCs release EVs containing microRNAs that are received by TCs, indicating a possible miR shuttle between TCs and stem cells in cardiac tissue. Moreover, we took advantage of the demonstration of TCs in bone marrow 23 and demonstrate that TC – derived microRNAs are received also by other types of stem cells, such as hematopoietic stem cells. Overall, these findings suggest that the miR – mediated paracrine signalling between TCs and stem cells is a general mechanism that occurs in various types of tissues.

## Materials and methods

### Cell cultures and animal models

**Fig 1:**
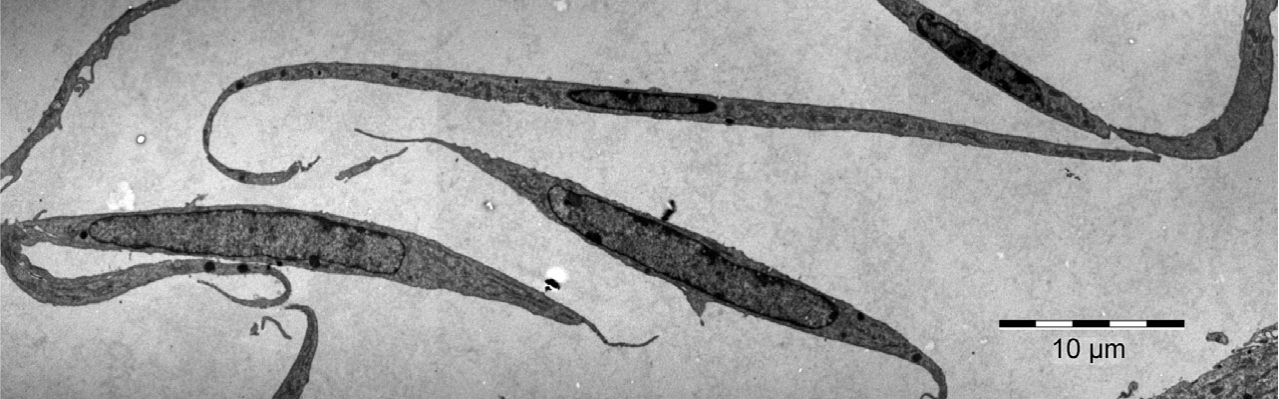
Rat cultured cardiac telocytes. Transmission electron microscopy image.

The TCs were isolated from hearts originating from 2 to 4-month old mice *C57BL/6J* (# 000664; The Jackson Laboratory, Bar Harbor, ME, USA) or dTomato expressing mice *ROS* ^*mT/mG*^ (# 007676; The Jackson Laboratory) and cultured as described elsewhere 13,30. Rat cardiac TCs were also isolated from 3-month old Wistars and cultured using the same procedure (Fig.1). All laboratory animals were used with the approval of the Institutional Ethical Committee.

**Fig 2:**
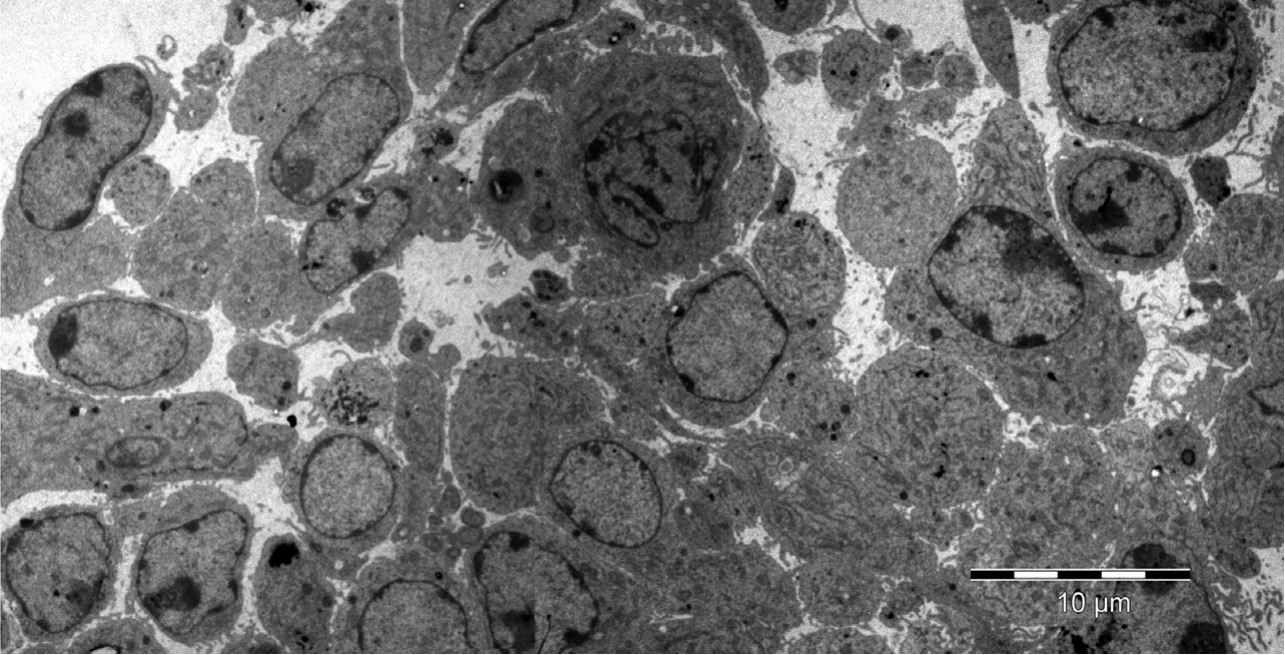
Rat cultured cardiac stem cells. Transmission electron microscopy (cultured cells were kindly provided by Prof P. Anversa, Harvard Medical School, Boston, MA, USA). The image was done by Dr. M Gherghiceanu, National Institute of Pathology, Bucharest, Romania.

*The rat CSCs* described elsewhere 31,32 *were a generous gift from Prof. Piero Anversa* (Brigham and Women Hospital, Boston, MA, USA). Briefly, these cells were plated at 10^5^ cells/25 cm^2^ and the culture was splitted at 60–75% confluence (Fig.2).

The hematopoietic stem and progenitor cells were isolated from the bone marrow of 2-month old mice *C57BL/6J* (# 000664; The Jackson Laboratory, Bar Harbor, ME, USA) by magnetic activated cell sorting (MACS) with anti-cKit coupled magnetic beads in accordance with manufacturer protocols (Miltenyi Biotec, Bergisch Gladbach, Germany). The MACS enriched cells were cultured with complete RPMI medium supplemented with 10% foetal bovine serum and with a standard antibiotic cocktail.

### Cell transfections and vesicle transfer assay

The subconfluent culture was incubated with the calcein AM dye (0.5 μg/ml; Merck KGaA, Darmstadt, Germany) for 30 min. in serum-free medium at 37°C. Then, the cells were washed extensively with complete medium and cultured for 24 hrs. Next day, the supernatant (conditioned medium, CM) of the donor culture was collected and centrifuged to remove floating cells and cell debris. The EV receptor cells were incubated with the CM for 16–24 hrs after which fluorescence was evaluated using the flow cytometer FACSCanto II (Becton Dickinson and Company, Franklin Lakes, NJ, USA).

For the microRNA transfer assay, a RNA oligo labelled with Cy5 that mimic miR-21 was used (Sigma-Aldrich, St. Louis, MO, USA). The EV donor cells were transfected with a mixture of RNA oligo and lipofectamine RNAiMAX (# 13778-075; Thermo Fisher Scientific, Waltham, MA, USA) in culture medium supplemented with 2% FBS. After 4–5 hrs, the culture was washed 5 times with fresh medium and incubated further with complete medium. Next day, the culture supernatant (CM) was centrifuged to remove floating cells and cell debris. The EV receptor cells were incubated with the CM for 16–24 hrs followed by fluorescence assessment using a flow cytometer FACSCanto II (Becton Dickinson and Company). The miR transfer through EVs was quantified as the percentage of receptor cells positive for Cy5. In some experiments, the CM was incubated with RNase A for degradation of any RNA fragments outside of EVs.

The immunophenotype of hematopoietic cells was determined by cell staining with the following antibodies: anti-cKit APC-Cy7 (clone 2B8; Biolegend, San Diego, CA, USA), anti-Sca1 PB (clone E13-161.7; Biolegend), anti-CD135 (Flt3) PE (clone A2F10; Biolegend), anti-CD150 Brilliant Violet™ 510 (clone TC15-12F12.2; Biolegend), biotinylated anti lineage panel (# 133307; Biolegend), Streptavidine PE-Cy5 (# 554062; Becton Dickinson and Company). Unspecific staining was avoided using the FC block kit (# 130-092-575; Miltenyi Biotec).

### Electron microscopy of cell cultures

Cultured cardiac TCs and cultured CSCs (kindly provided by Prof. Anversa, as mentioned above) were studied by transmission electron microscopy as described previously 13,29. Cells were fixed in 0.1 M cacodylate buffer (pH 7.4) with 2.5% glutaraldehyde and 1.4% sucrose, at 37°C for 5 min. Cells were scraped, resuspended in the same fixative for 4 hr at 4°C and then post-fixed for 1 hr in buffered 1% OsO_4_ with 1.5% K_4_Fe(CN)_6_ (potassium ferrocyanide-reduced osmium). Fixed cells were spun at 850 × g, embedded in 1% agar gel, and further processed for final epoxy resin embedding. The ultra-thin sections were cut using a diamond knife and, double stained with 1% uranyl acetate and Reynolds lead citrate. The 60 nm thin sections were visualized using a Morgagni 268 TEM (FEI Company, Eindhoven, The Netherlands) at 80 kV. Digital electron micrographs were recorded with a MegaView III CCD and images processed using iTEM-SIS software (Olympus, Münster, Germany). Figure1 shows cardiac TCs in culture and Figure2 shows cultured CSCs as determined by transmission EM.

### Statistical analysis

Data are presented as means ± SD. One-way (anova) and the LSD tests were applied using SPSS version-11. *P* < 0.05 was considered statistically significant.

## Results

### Telocytes release and receive extracellular vesicles in culture

**Fig 3:**
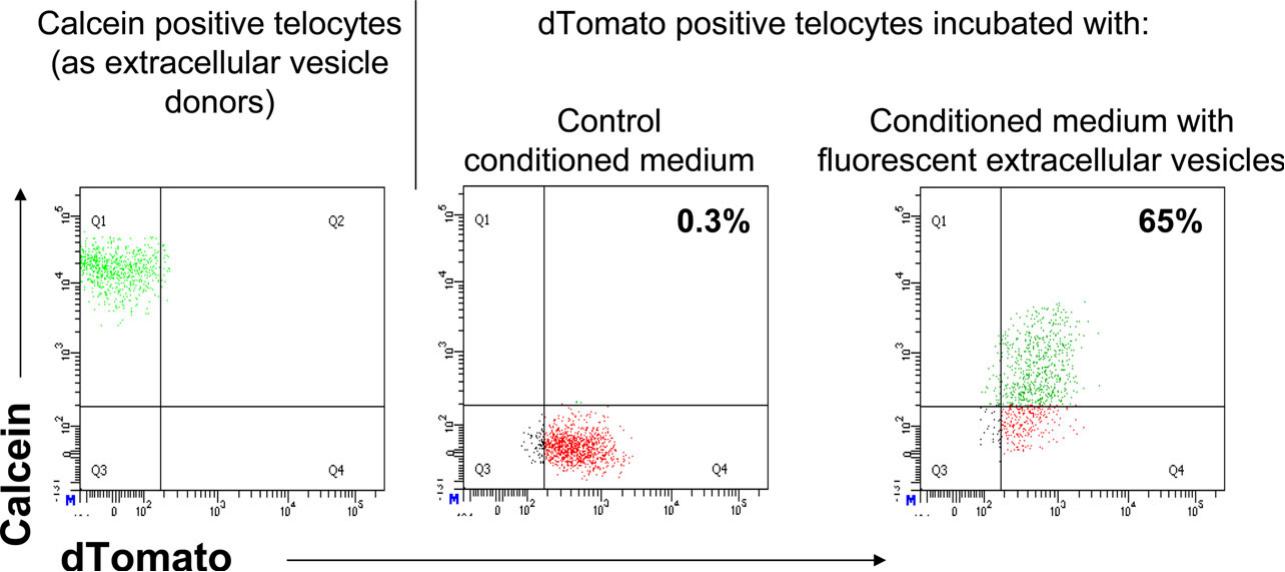
Mouse telocytes release and receive extracellular vesicles. Extracellular vesicle donor telocytes are negative for red fluorescent protein, dTomato (left panel), while extracellular vesicle receptor telocytes are positive for red fluorescence (middle and right panels). In left panel, extracellular vesicle donor telocytes are green fluorescent after incubation with calcein. Extracellular vesicle receptor telocytes are green fluorescent only after incubation with medium conditioned by calcein positive, extracellular vesicle donor telocytes (right panel), but not in case of conditioned medium derived from calcein negative cells (left panel).

Our previous data indicated that TCs release EVs in culture 29. We evaluated if the EVs can be endocyted by other TCs and thus contribute to the paracrine signalling in the TC network. Calcein was used as tracking label for EV transfer. The donor cells (TCs) were incubated with the liposoluble form of calcein (calcein AM). Inside of donor cells, calcein AM is converted to the green fluorescent form. Figure3, left plot shows that TCs incubated with calcein becomes positive for calcein fluorescence. The fluorescent form is not liposoluble and thus remains trapped within any membrane bounded structure, such as EVs. In this set up, the donor cells labelled with calcein should release fluorescent EVs in the culture medium. To test if these fluorescent EVs are endocyted by other TCs, the CM was incubated with a culture of TCs that express dTomato (red). Thus, the EV receptor and donor TCs are distinguished based on the presence or absence of dTomato fluorescence, respectively. Calcein was detected in EV receptor TCs only when dTomato positive cells were incubated with the culture medium conditioned by fluorescent EV releasing culture (Fig.3, right plot) but not in case of CM derived from the control culture (Fig.3, middle plot).

### Telocytes transfer microRNAs to hematopoietic stem cells by extracellular vesicles

**Fig 4:**
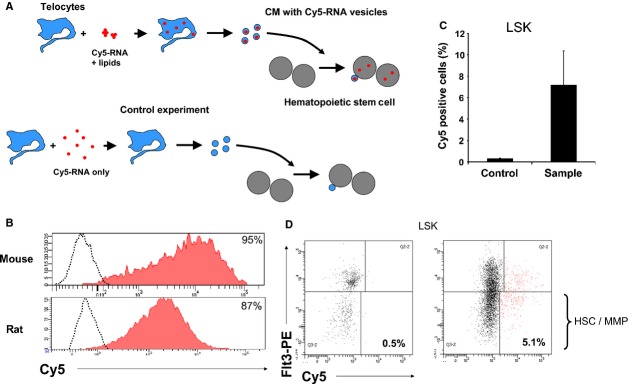
Hematopoietic stem cells receive microRNA loaded extracellular vesicles released by Cy5-RNA positive telocytes. (A) *Experimental set-up*: Cardiac telocytes are transfected with lipofectamine/Cy5-RNA complexes and release extracellular vesicles loaded with fluorescent-RNA into culture supernatant; hematopoietic stem cells incubated with supernatant receive the Cy5-RNA vesicles and become fluorescent; in control experiments, telocytes are not transfected with oligoRNA in absence of lipofectamine, the released vesicles does not have Cy5-RNA and the corresponding hematopoietic stem cells are negative for Cy5 fluorescence. (B) *TC transfection with Cy5-RNA*: Rat or mouse telocytes are positive for fluorescent Cy5 after 3–5 hrs incubation with a mixture of Cy5-RNA oligo and Lipofectamine RNAiMAX (red filled areas). Controls, non-fluorescent telocytes, represent cells incubated with oligoRNA in absence of lipids (dot plots). (C) *Cy5-RNA positive hematopoietic stem/progenitor cells*: Hematopoietic progenitors (LSK) are fluorescent after incubation with supernatant derived for Cy5-RNA transfected telocytes (sample), but not in case of supernatant derived from telocytes negative for Cy5 (control). LSK cells Lineage/7AAD negative, Sca1 positive and c-Kit positive. *N* = 4, *P* = 0.03 (D) *Representative FACS plots of data presented in (C)*. Right plot: Hematopoietic stem cells (HSC) and their multipotent progeny (MPP), defined as cells LSK Flt3 negative, are positive for Cy5 when cultured in presence of medium conditioned by Cy5-RNA transfected telocytes. Left plot: in control experiments, HSC/MPP have been incubated with conditioned supernatant derived from non-fluorescent telocytes.

Since TCs are widely distributed within the stroma of many organs and are part of stem cell niche, we raised the question whether the TC derived EVs could be endocyted by the stem cells. For this purpose we chose the hematopoietic stem cells as recipient cells because these are very well characterized immunophenotipically and because TCs are found in bone marrow 23. Moreover, we tested if the EVs released by TCs contain microRNAs that can be thus delivered to stem cells. The experimental set-up is described in Figure4A. To track microRNAs we have used oligoRNA labelled with Cy5 that mimics miR-21. Flow cytometry data indicated that more than 80% of TCs were successfully transfected with miR oligos after being incubated with a mixture of Cy5-RNA and lipids (Fig.4B). The fluorescent signal is specifically derived from intracellular Cy5-RNA since there are no fluorescent TCs in absence of lipids in control experiments (Fig.4B). Transfection efficiency was high regardless of the species from which TCs originated (mouse or rat). The transfected TCs (Cy5 fluorescent or control cells) release EVs into culture supernatant. The hematopoietic stem and early progenitors are defined as cells negative for lineage markers, Sca-1 positive and cKit positive (population LSK). Figure4C shows that the LSK population became Cy5 positive after 24 hr incubation with culture medium conditioned with fluorescent TCs but not in case of CM derived from control cultures. Since the control is represented by donor cells treated with Cy5-RNA oligos in absence of lipofectamine, the possibility that fluorescent LSK are labelled unspecifically with free Cy5-RNA oligos carried by CM was excluded. Within LSK population, the stem cells are defined as cells positive for CD150 and negative for CD135 (Flt3) 33. Since the expression of CD150 is lost during culture (data not shown), we have analysed the cells negative for CD135. The population LSK CD135 negative includes both HSC and multipotent hematopoietic progenitors (MPP) 33. Figure4D shows that 5% of the HSC/MPP are positive for Cy5 fluorescence proving for a miR-21 mimic oligo transfer between TCs and hematopoietic cells. By contrast, no signal was detected in case of HSC/MPPs incubated with culture medium conditioned with control cells. Overall the data suggest that TCs regulates the posttranscriptional machinery of stem cells by microRNAs delivered with EVs.

### MicroRNA exchange between telocytes and cardiac stem cells

**Fig 5:**
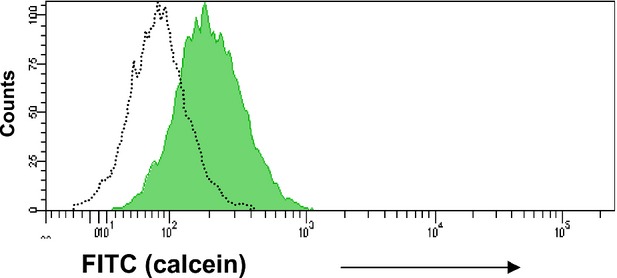
Cardiac stem cells receive fluorescent extracellular vesicles released by calcein positive telocytes. Rat cardiac stem cells are FITC positive (grey area) after incubation with culture media conditioned by calcein positive cardiac telocytes. The control (dot line) represent cardiac stem cells incubated with supernatant derived from calcein negative telocytes. The figure is a representative histogram of 4 independent experiments.

**Fig 6:**
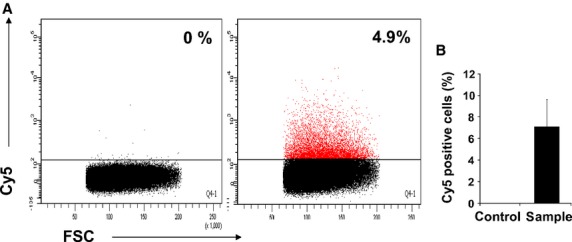
Cardiac stem cells receive microRNA loaded extracellular vesicles released by Cy5-RNA positive telocytes. (A) Rat cardiac stem cells are Cy5 positive after incubation with supernatant derived from fluorescent telocytes (right plot). In the control experiment, cardiac stem cells were incubated with supernatant derived from Cy5-RNA free telocytes (left plot). The figure shows representative data of 4 independent experiments. (B) Statistical data of 4 experiments (*T* = 0.02).

We have evaluated if microRNA loaded EVs generated by TCs are received by stem cells found in cardiac tissue 9,32. Figure5 shows that calcein is detected in CSCs by flow cytometry when the cells are incubated with the CM derived from fluorescent donor cultures. The data indicates that TCs release EVs that are taken up by CSC *via* endocytosis *in vitro*. When the EV donor TCs are transfected with the fluorescent miR-21 mimic oligo, the CSCs are Cy5 positive after incubation with the corresponding CM (Fig.6). Moreover, the recipient CSC are positive for Cy5 when incubated with the supernatant in presence of RNase A, which likely degraded any free RNA fragments present in solution (data not shown). Overall, our data strongly indicates that *TCs deliver microRNAs to CSC by means of EVs*.

**Fig 7:**
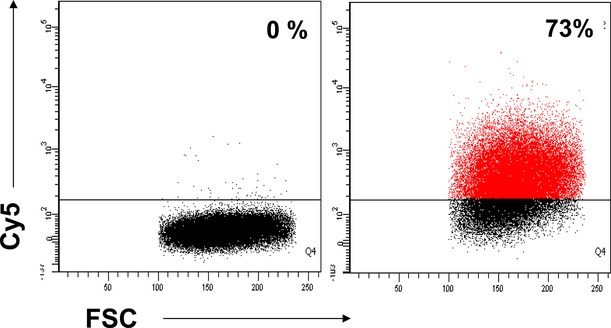
Telocytes receive microRNA loaded extracellular vesicles released by Cy5-RNA positive cardiac stem cells. Cardiac telocytes are Cy5 positive after incubation with culture supernatant derived from fluorescent cardiac stem cells (right plot). In the control experiment, telocytes were incubated with supernatant derived from Cy5-RNA free cardiac stem cells (left plot). The figure shows representative data of 2 experiments.

It was shown that mesenchymal stem cells release vesicles (microparticles) loaded with microRNAs, either as precursors or in mature form 34,35,6. Therefore, we have asked if CSCs can signal back to TCs with miR containing EVs. Figure7 shows that cardiac TCs uptake Cy5-RNA after incubation with the culture medium conditioned by fluorescent transfected CSCs. Overall the data suggest the presence of a microRNA shuttle between TCs and CSC in cardiac tissue.

## Discussion

Intercellular communication is essential for tissue to act as a functional unit. Paracrine signalling is part of this communication ensuring that cells of the same type distributed within the tissue functional unit will respond in an identical way. As a result of their morphological characteristics, TCs are likely to be involved in tissue homeostasis as a coherent unit and several studies support the view that TCs act as an integrative network of immune, nervous and endocrine signals 20. Generally speaking at a tissue level TC establish two types of activities: long distance *via* Tps, and short distance *via* EVs. To accomplish this role, TCs release EVs *in situ* from their body and along of their Tps 29. It has been shown that TCs release three types of EVs in culture: exosomes, ectosomes and multivesicular cargos 29. It is expected that TCs derived EVs have a similar composition with the ones released by other cell types, namely soluble factors, RNA and proteins and the identification of these factors may offer better clues regarding the TCs paracrine signalling (www.exocarta.org). Here, we show that TCs release and internalize EVs loaded with microRNAs influencing the post-transcriptional machinery and cells respond faster, eventually.

Since the demonstration that EVs carry mRNAs and microRNAs and that the microRNA effector complexes are coupled with multivesicular bodies it becomes clear that EVs transfer epigenetic signals between cells 5,36,37. Here, we show that TCs employ such mechanisms to regulate surrounding tissue including stem cells.

Several groups have shown that the EVs released by cardiospheres, mesenchymal stem cells or cultured cardiac stroma play protective roles after myocardial infarction by stimulating cell proliferation and inhibiting apoptosis and microRNAs are an important component of this anti-apoptotic programme 8,34,38–40. Published papers support the hypothesis that TCs have a role in cardiac tissue regeneration and the data presented here reinforce this idea 20.

Bidirectional signalling between adult stem cells and the surrounding tissue contributes to the homeostasis of the stem cell population and consequently to the regenerative needs of the tissue. In steady-state conditions, most of the stem cell population is maintained in a non-proliferative phase (dormant) within the stem cell niche 41,42. The regulatory signal network that controls the balance between the dormant and proliferative states of stem cells is not fully understood, but a growing body of data demonstrates that several types of cells have specific or overlapping contributions to stem cell homeostasis 43. Most of regulatory cells are part of the stem cell niche. TCs were found in CSC niche where establish direct contacts with both the putative stem cells and cardiomyocyte progenitors 12,21,44,45. The stem cell niche location of TCs is demonstrated for various tissues and organs, such as iris stroma, lung, skin, skeletal muscle, choroid plexus 25,45. Our data suggest that TCs regulates stem cells not only by direct intercellular contacts (junctions) but also indirectly through EVs. We provide evidence that TCs have an epigenetic control to stem and progenitor cells suggesting a contribution of TCs in regulation of both cardiac and hematopoietic tissue renewal. Depending of the type of miRs delivered by EVs, TCs might contribute to the balance between quiescent and proliferative states and between stem cell self-renewal and differentiation. Adult stem cells release EVs 34,35,6 indicating that the vesicle mediated signalling between stem and niche is bidirectional and our data bring support to this view.

Last but not least, several authors 46–48 assumed recently that TCs might act as a primitive ‘local’ nervous system for the cells found in close relationships with TCs. Smythies and Edelstein 47,48 suggest that TCs are well equipped (EV release, intercellular junctions, ion channels) to play at least a part of the role needed in a bioelectric information pathway described by Levin 46.

**Fig 8:**
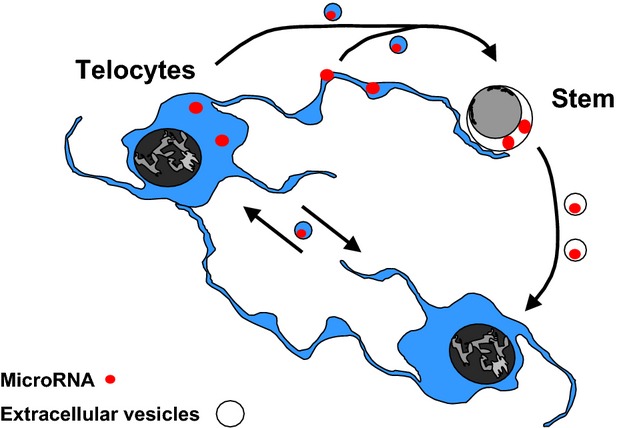
Telocytes and stem cells shuttle microRNA by extracellular vesicles.

*In conclusion*, our results show that TCs in various tissues (including myocardium) deliver microRNAs to stem cells by means of EVs. Moreover, the epigenetic signalling between TCs and stem cells seems to be bidirectional (reciprocal, Fig.8).
